# The Brain Network of Naming: A Lesson from Primary Progressive Aphasia

**DOI:** 10.1371/journal.pone.0148707

**Published:** 2016-02-22

**Authors:** Raffaella Migliaccio, Claire Boutet, Romain Valabregue, Sophie Ferrieux, Marie Nogues, Stéphane Lehéricy, Didier Dormont, Richard Levy, Bruno Dubois, Marc Teichmann

**Affiliations:** 1 Institut du Cerveau et de la Moelle Epinière, UMR INSERM-CNRS-UPMC 1127, Frontlab, Paris, France; 2 Department of Neurology, National Reference Center for « PPA and rare dementias », Pitié Salpêtrière Hospital, AP-HP, Paris, France; 3 Centre de Neuro-imagerie de Recherche (CENIR), Institut du Cerveau et de la Moëlle Epinière, Paris, France; 4 Université Pierre et Marie Curie, INSERM, UMR-S 678, Paris, France; 5 Service de Neuroradiologie Diagnostique et Fonctionnelle, Hôpital de la Pitié-Salpêtrière, AP-HP, Paris, France; 6 Department of Neurology, Hôpital Saint Antoine, AP-HP, Paris, France; IIBB/CSIC/IDIBAPS, SPAIN

## Abstract

**Objective:**

Word finding depends on the processing of semantic and lexical information, and it involves an intermediate level for mapping semantic-to-lexical information which also subserves lexical-to-semantic mapping during word comprehension. However, the brain regions implementing these components are still controversial and have not been clarified via a comprehensive lesion model encompassing the whole range of language-related cortices. Primary progressive aphasia (PPA), for which anomia is thought to be the most common sign, provides such a model, but the exploration of cortical areas impacting naming in its three main variants and the underlying processing mechanisms is still lacking.

**Methods:**

We addressed this double issue, related to language structure and PPA, with thirty patients (11 semantic, 12 logopenic, 7 agrammatic variant) using a picture-naming task and voxel-based morphometry for anatomo-functional correlation. First, we analyzed correlations for each of the three variants to identify the regions impacting naming in PPA and to disentangle the core regions of word finding. We then combined the three variants and correlation analyses for naming (semantic-to-lexical mapping) and single-word comprehension (lexical-to-semantic mapping), predicting an overlap zone corresponding to a bidirectional lexical-semantic hub.

**Results and Conclusions:**

Our results showed that superior portions of the left temporal pole and left posterior temporal cortices impact semantic and lexical naming mechanisms in semantic and logopenic PPA, respectively. In agrammatic PPA naming deficits were rare, and did not correlate with any cortical region. Combined analyses revealed a cortical overlap zone in superior/middle mid-temporal cortices, distinct from the two former regions, impacting bidirectional binding of lexical and semantic information. Altogether, our findings indicate that lexical/semantic word processing depends on an anterior-posterior axis within lateral-temporal cortices, including an anatomically intermediate hub dedicated to lexical-semantic integration. Within this axis our data reveal the underpinnings of anomia in the PPA variants, which is of relevance for both diagnosis and future therapy strategies.

## Introduction

Patients with primary progressive aphasia (PPA) usually report difficulty in word finding as the first and main complaint. Such difficulty, termed anomia, can manifest in both, spontaneous speech or picture naming and is thought be the “single most common sign of PPA” [1]. According to language models, word finding is a multi-step process essentially involving access to the words’ semantics and subsequent lexical retrieval of the phonological code [2]. These two core steps appear to depend on anterior and posterior regions of the temporal cortex [3–5]. However, the use of various degenerative and vascular lesion models has led to divergent findings involving various temporal, parietal and frontal regions [6], thus resulting in an unsolved debate. In addition, several authors have suggested integrative brain regions, labeled ‘convergence zones’ [7, 8], which might bind semantic and lexical information to ultimately form the basis for the phonological spell-out of articulated words. However, it is unclear whether there is a unique integrative hub underpinning one core operation, i.e. ‘binding’, and where such a hub might be localized. An important challenge is therefore to clarify the current situation with a single lesion model encompassing the entire language cortex with the aim of disentangling the core regions of naming and identifying a potential integrative hub. PPA represents such a model providing a unique setting where the different components of the language network undergo progressive and selective dissolution related to neural degeneration. Unlike vascular models with stroke patients, which are anatomically restricted due to the blood vessel distribution [9, 10], PPA affects the whole range of language-related cortical regions [11–13].

PPA is subdivided into three main variants (logopenic (lv-PPA), semantic (sv-PPA) and agrammatic/nonfluent (nfv-PPA), affecting posterior temporal-parietal, anterior temporal and inferior frontal/superior temporal cortices, respectively [11]. Such distinct atrophy patterns suggest that the naming disorders in the three PPA variants are related to the breakdown of distinct and anatomically separable components of the lexical/semantic processing system. However, the substrates of naming deficits in PPA have not been directly compared between the three main variants given that the few available studies did not explore the whole range of PPA [6], lumped PPA variants together [14], or mixed PPA with other degenerative conditions such as Alzheimer’s disease or frontotemporal dementia [15, 16]. In addition, the early occurrence of word finding difficulties in all PPA variants [1] might suggest the existence of common damage to a unique central hub subserving the integration of semantic and lexical word information. According to models of word production such a hub would probably correspond to the intermediate level of ‘lexical selection’ or ‘lemmas’, mediating between ‘conceptual preparation’ and the activation of ‘word forms’ within the mental lexicon [2, 17]. These models also claim that the intermediate level is shared for word production and comprehension given the consistent interference effects between auditorily perceived distractors (e.g., ‘goat’) and the production of related target words during naming (e.g., ‘sheep’) [18]. However, the localization of a bidirectional lexical-semantic hub has not been clarified and its alteration in PPA has not been investigated.

With the aim of addressing these issues the present study had three interrelated goals: i) to determine the core regions impacting naming in PPA variants, ii) to disentangle the different components of the word finding process, and iii) to identify a potential lexical-semantic hub. For this purpose we explored a large group of patients with sv-PPA, lv-PPA and nfv-PPA assessing their performances in a picture naming task and identifying patterns of cortical atrophy by using voxel based morphometry (VBM). We then analyzed correlations between behavioral scores and cortical atrophy for each PPA variant to identify the regions impacting naming in PPA and to disentangle the core regions of the word finding process. In a second series of correlation analyses, we combined the three PPA subgroups with the aim of identifying a unique and anatomically distinct region impacting naming in all PPA variants and potentially corresponding to a central hub for lexical/semantic integration. We then checked the claim that such a hub should be bidirectional, linking both, semantic to lexical and lexical to semantic representations [2, 7, 8, 17]. To confirm this double function we compared ‘picture naming’, requiring semantic-to-lexical mapping, and ‘single-word comprehension’, requiring lexical-to-semantic mapping. We expected to identify a common region for both tasks, corresponding to the core of the integrative lexical-semantic hub which was thought to be localized in mid portions of the left temporal cortex.

## Methods

### Participants

Thirty PPA patients were enrolled in the study at the National Reference Center for “PPA and rare dementias” of the Pitié-Salpêtrière Hospital, Paris. Clinical diagnosis was based on a multi-disciplinary evaluation including neurological examination, standard neuropsychological tests and a detailed language evaluation. Diagnosis was based on current research criteria [19], in which progressive language impairment is required as the central core. The patients were further classified into the three PPA main variants: 12 patients had sv-PPA, 11 patients lv-PPA, and 7 patients nfv-PPA. According to the classification criteria [19], sv-PPA patients had single-word comprehension deficits and anomia (without sentence repetition disorders, agrammatism or motor speech disorders), lv-PPA patients were characterized by word finding difficulties and sentence repetition impairment (without agrammatism, single-word comprehension or motor speech disorders), and patients with nfv-PPA demonstrated speech sound errors and/or syntactic disorders in sentence production/perception (without single-word comprehension disorders). Patients were excluded if they had: (i) medical illnesses that could interfere with cognitive functioning; (ii) any other major systemic, psychiatric or neurological diseases; and (iii) non degenerative lesions on routine MRI such as focal or diffuse brain damage including cerebrovascular disorders.

All clinical and imaging data were generated during a routine clinical work-up and were retrospectively extracted for the purpose of this study. Therefore, according to French legislation, explicit consent was waived. However, regulations concerning electronic filing were followed, and patients and their relatives were informed that anonymized data might be used in research investigations and particularly for the present study. The study received approval from the Ethics Committee of the Pitié Salpêtrière Hospital (Paris, France), and patient information was anonymized and de-identified prior to analysis. Demographic data of the patients are summarized in Table 1.

**Table 1:** Demographic patient data (means ± standard deviations).

	all PPA	nfv-PPA	lv-PPA	*sv-PPA*
**Number of subjects**	30	7	12	*11*
**Women/men**	14/16	5/2	4/8	*5/6*
**Mean age (years)**	67 ± 7.7	70.3 ± 5.8	68.9 ± 8.2	*62*.*6 ± 6*.*6*
**Right/left handed**	27/3	7/0	11/1	*9/2*
**Education level (years)**	10.4 ± 3.7	9.9 ± 4.0	10.2 ± 3.6	*11 ± 3*.*9*
***Symptom duration***	*3*.*2 ± 1*.*2*	*3*.*1 ± 1*.*3*	*3*.*2 ± 0*.*58*	*3*.*4 ± 1*.*6*

nfv = non fluent variant; lv = logopenic variant; sv = semantic variant

### Cognitive/language assessment

The core of the language assessment was composed of a picture naming test (D080 [20]) and a single-word comprehension task requiring pointing to pictures upon auditory word presentation (Boston Diagnostic Aphasia Evaluation [21]). The DO80 contained 80 pictures depicting nouns (mean lexical frequency 34 ± 59 per million according to the LEXIQUE 2 database [22]. Patients were asked to name aloud each of the 80 items within 6 seconds after the picture onset. The rating of naming performance explicitly focused on lexical/semantic core mechanisms of the word finding process accepting post-lexical phoneme substitutions and phonetic distortions whenever the target word was recognizable. Answers were therefore counted as correct when phonemic paraphasias approximated the word target as for example in ‘edephant’ for ‘elephant’. In contrast, semantic paraphasias (e.g., ‘cow’ instead of ‘elephant’), non responses, unrelated words and unrecognizable nonce word productions were counted as errors. Both key tests of this study (DO80, single-word comprehension of the BDAE) were used because they offer a wide range of stimuli (nouns, numbers, living and non living items) and therefore provide global markers for naming and word comprehension without restricting the materials to one word or semantic category.

The language assessment furthermore included an evaluation of aphasia severity taking into account spontaneous speech and the description of the ‘cookies theft picture’ (Boston Diagnostic Aphasia Evaluation [21]), a verbal fluency test comprising phonemic and category fluency [23], and a sentence repetition task (Boston Diagnostic Aphasia Evaluation [21]. We also evaluated syntax applying the 4-point scale of Leyton et al. [24] scoring “severe” (3), “mild” (2), “questionable” (1) and “no” (0) agrammatism during spontaneous speech, a ten-minute conversation, and during the description of the ‘cookies theft picture’. Motor speech errors (phonetic/speech sound distortions) were assessed during the DO80 picture naming test. Global cognitive assessment included the Mini-Mental State Examination [25] and the Frontal Assessment Battery [26].

As all standard language/cognitive tests can be biased by visuo-perceptual disorders we also applied a subtest of the PEGV (Protocole Montréal Toulouse d’Evaluation des Gnosies Visuelles) [27]: pointing to specific geometric shapes contained in a setting of entwined geometrical shapes, with the aim to probe for accurate performance in the perceptual domain.

### Imaging study

#### MRI acquisition and voxel-based morphometry study

Three-dimensional T1 weighted MRIs were acquired on a 1.5 T scanner (GE Medical Systems GE Healthcare, Little Chalfont, UK). Voxel-based morphometry (VBM) was performed using the Statistical Parametric Mapping software (SPM8, Welcome Department of Imaging Neuroscience, London; http://www.fil.ion.ucl.ac.uk/spm) running on Matlab 7.13.0 (Math-Works, Natick, MA), and the Diffeomorphic Anatomical Registration Exponentiated Lie Algebra (DARTEL) registration method [28]. Images were spatially normalised into the Montreal Neurological Institute (MNI) space and then segmented into grey matter GM, white matter and CSF. The images were also modulated, and we chose the option “non-linear only”, in which voxel values are multiplied by non-linear components, which allows the absolute amount of tissue corrected for individual brain sizes to be considered, without entering the total intracranial volume as a covariate in the statistical model. Gray matter was further normalized with DARTEL (http://www.fil.ion.ucl.ac.uk/spm/software/spm8). Normalized segments were smoothed with an 8-mm full-width at half-maximum Gaussian kernel.

In order to define the pattern of gray matter atrophy specific to each PPA variant, we performed contrasts comparing each PPA variant *vs*. the other two PPA subgroups (e.g., sv-PPA vs. lv-PPA and nfv-PPA). A significance threshold of p < 0.001 uncorrected for multiple comparisons was accepted given the relatively small sample size of the subgroups, and because contrasts were performed between patients.

The relationship between performances on language tests and gray matter volume was analyzed both, by exploring each group separately and by collapsing across subject groups. We investigated the effect of each variable (‘picture naming’, ‘single-word comprehension’) separately, by using a multiple regression design, with age and disease duration as nuisance variables and separate design matrices for each test. Analyses were focused on the left hemisphere. A significance threshold of p < 0.05 corrected for multiple comparisons (Family Wise Error [FWE]) was accepted for all PPA patients. Results were also tested at p < 0.001 uncorrected for multiple comparisons when exploring each patient group separately. This threshold was accepted given the relatively small sample size of the subgroups and because the literature indicates that lexical-semantic processing is primarily linked to a particular brain region: the temporal cortex [3–5].

## Results

### Cognitive/language assessment

We applied ANOVAs to compare the three PPA subgroups. These subgroups were similar with respect to age, gender distribution, handedness, number of years of education and symptom duration (all Fs < 1). Performance with the MMSE and the FAB was poorer in lv-PPA than in sv-PPA and nfv-PPA (MMSE: lv-PPA vs. nfv-PPA F(1,17) = 12.10, p = 0.003; lv-PPA vs. sv-PPA F(1,21) = 11.05, p = 0.003; FAB: lv-PPA vs. nfv-PPA, F(1,17) = 5.86, p = 0.027; lv-PPA vs. sv-PPA F(1,21) = 4.66, p = 0.043). Aphasia severity was similar in the three PPA subgroups (F(2,27) = 1.63, p = 0.22). Table 2 summarizes the scores from the cognitive/language assessments. Detailed results of pair-wise comparisons between subgroups for the different language tests are shown in Table 3.

**Table 2:** Cognitive/language data of the patients (means ± standard deviations).

	all PPA	nfv-PPA	lv-PPA	sv-PPA	*Normal threshold*
MMSE	22.7 ± 4.8	26.1 ± 2.0	19.0 ± 5.2	24.6 ± 2.3	*≥ 27*
FAB	12.0 ± 3.1	13.6 ± 2.6	10.3 ± 2.9	12.9 ± 2.8	*≥ 16*
Aphasia severity scale	3.5 ± 0.9	3.1 ± 0.9	3.3 ± 0.8	3.8 ± 0.9	*> 4*
**Picture naming**	56.0 ± 21.2	75.9 ± 5.0	60.8 ± 14.4	38.1 ± 20.1	*> 75*
Non responses	12.3 ± 15.0	0.9 ± 1.5	10.6 ± 10.7	21.5 ± 18.4	*NA*
Phonemic paraphasias	1.4 ± 1.9	3.6 ± 2.5	0.8 ± 1.2	0.5 ± 0.8	*NA*
Semantic paraphasias	7.3 ± 7.9	0.3 ± 0.5	3.9 ± 2.0	15.5 ± 7.2	*NA*
Phonetic distortions	2.2 ± 4.3	9.3 ± 3.3	0 ± 0	0 ± 0	*NA*
**Single-word comprehension**	64.8 ± 9.5	71.3 ± 0.8	67.8 ± 6.7	57.4 ± 10.8	*≥ 68*
**Category fluency** (‘fruits’ per 2 minutes)	9.6 ± 5.1	15.6 ± 4.8	8.3 ± 3.7	7.5 ± 3.7	*≥ 15*
**Phonemic fluency** (‘P’ per 2 minutes)	9.4 ± 5.6	10.7 ± 7.8	7.8 ± 4.5	10.2 ± 5.3	*≥ 15*
**Sentence repetition**	9.8 ± 4.0	6.9 ± 3.1	8.6 ± 3.3	13.1 ± 3.0	*≥ 13*
***Syntax scores***	*0*.*6 ± 1*.*1*	*2*.*4 ± 0*.*5*	*0*.*1 ± 0*.*3*	*0 ± 0*	*< 1*

nfv = non fluent variant; lv = logopenic variant; sv = semantic variant; MMSE = mini mental state examination; FAB = frontal assessment battery; NA = not applicable. Syntax scores: based on the 4-point scale of Leyton et al. [24]: 3 = severe, 2 = mild, 1 = questionable, 0 = no agrammatism. Phonetic distortions: number of phonetic distortions during the picture naming test.

**Table 3:** Results of the language assessment: pair-wise ANOVA comparisons between PPA subgroups.

	lv-PPA *vs*. nfv-PPA	nfv-PPA *vs*. sv-PPA	lv-PPA *vs*. sv-PPA	*Inter-variant comparison*
**Picture naming** (total score)	F(1,17) = 6.93, p = 0.017*	F(1,16) = 23.37, p < 0.001*	F(1,21) = 9.86, p = 0.005*	*sv < lv < nfv*
Non responses	F(1,17) = 5.64, p = 0.03*	F(1,16) = 8.53, p = 0.01*	F(1,21) = 3.07, p = 0.09	*nfv < lv = sv*
Phon paraphasias	F(1,17) = 10.56, p = 0.005*	F(1,16) = 14.10, p = 0.002*	F < 1	*sv = lv < nfv*
Sem paraphasias	F(1,17) = 21.38, p < 0.001*	F(1,16) = 30.99, p < 0.001*	F(1,21) = 29,23, p < 0.001*	*nfv < lv < sv*
Phonetic distor	F(1,17) = 59.60, p < 0.001*	F(1,16) = 93.04, p < 0.001*	F < 1	*nfv < lv = sv*
**Single-word comprehension**	F(1,17) = 3.07, p = 0.09	F(1,16) = 11.19, p = 0.004*	F(1,21) = 6.37, p = 0.02*	*sv < lv = nfv*
Category fluency	F(1,17) = 14.09, p = 0.002*	F(1,16) = 16.43, p = 0.001*	F < 1	*sv = lv < nfv*
Phonemic fluency	F(1,17) = 1.06, p = 0.32	F < 1	F(1,21) = 1.32, p = 0.26	*sv = lv = nfv*
Sentence repetition	F(1,17) = 1.27, p = 0.28	F(1,16) = 18,25, p = 0.001*	F(1,21) = 11.78, p = 0.002*	*nfv = lv < sv*
*Syntax scores*	*F(1*,*17) = 157*.*12*, *p < 0*.001*	*F(1*,*16) = 235*.*48*, *p < 0*.001*	*F < 1*	*nfv < lv = sv*

nfv = non fluent variant; lv = logopenic variant; sv = semantic variant; Phon: Phonemic; Sem: semantic; Phonetic distor = phonetic distortions. ‘<‘ means poorer scores or frequency; ‘ = ‘ means similar scores or frequency.

Picture naming scores were abnormal in 83.3% of the patients (sv-PPA and lv-PPA 100% impaired, nfv-PPA 28.6% impaired). At a group level, naming performance was poorer in sv-PPA than in lv-PPA, and poorer in lv-PPA than in nfv-PPA. Phonemic paraphasias were more frequent in nfv-PPA than in sv-PPA and lv-PPA, whereas semantic paraphasias were more frequent in sv-PPA than in lv-PPA and more frequent in lv-PPA than in nfv-PPA. Non responses were more frequent in sv-PPA and lv-PPA than in nfv-PPA. In the single-word comprehension task performance was poorer in sv-PPA than in lv-PPA and nfv-PPA. Mean scores of word comprehension in the lv-PPA group were slightly under the cut-off because two lv-PPA patients had mild word comprehension difficulties. Such difficulties are, however, not at odds with an lv-PPA diagnosis given that semantic disorders in lv-PPA have been detected in several studies [29, 30], and given that both patients met the lv-PPA core features of word-finding difficulties and sentence repetition impairment. Finally, category fluency was poorer in sv-PPA and lv-PPA than in nfv-PPA, phonemic fluency was similar in the three subgroups, and sentence repetition scores were poorer in lv-PPA and nfv-PPA than in sv-PPA.

Importantly, the results of the visuoperceptual subtest of the PEGV [27] showed normal performances in all PPA patients ensuring that language/cognitive data were not biased by ‘low level’ visuoperceptual dysfunction.

We also conducted correlation analyses between language scores in the whole PPA group using tasks tapping both lexical and semantic capacities, in word production and perception (‘picture naming’, ‘single-word comprehension’, ‘category fluency’). Results showed that picture naming correlated with single-word comprehension (R = 0.79, p < 0.001) and with category fluency (R = 0.66, p < 0.001), and that category fluency correlated with single word comprehension (R = 0.51, p = 0.004). These significant correlations between the three tasks suggested a common substrate for the concomitant processing of semantic and lexical word aspects in both, word production and perception. In contrast, phonemic fluency, which was used as a control test involving lexical but not semantic capacities, didn’t correlate neither with naming, single-word comprehension or category fluency.

### Imaging study

#### Atrophy patterns specific to each PPA variant

**Fig 1:**
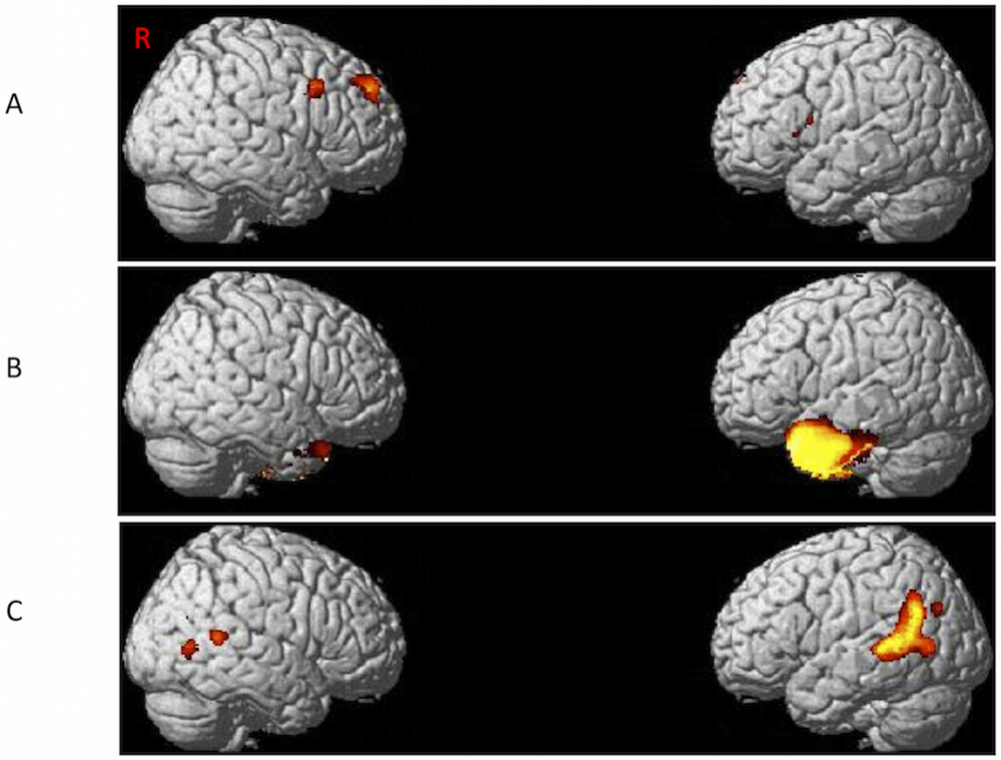
Regions of gray matter atrophy specific to each PPA variant as compared with other two variants. Regions of gray matter atrophy are shown on the 3-dimensional rendering of the Montreal Neurological Institute standard brain. A) nfv-PPA, B) sv-PPA, C) lv-PPA.

Gray matter atrophy specific to sv-PPA involved the whole left anterior temporal cortex. Smaller areas of atrophy were also found in the right anterior temporal cortex. Lv-PPA had variant-specific atrophy in the left temporal-parietal junction. Smaller areas of atrophy were also found in the right posterior superior and middle temporal gyrus. The atrophy pattern specific to nfv-PPA included the left inferior frontal gyrus as well as right superior and middle frontal gyri. Fig 1 illustrates the results of variant-specific atrophy patterns.

#### Correlations between language scores and gray matter volume

**Fig 2:**
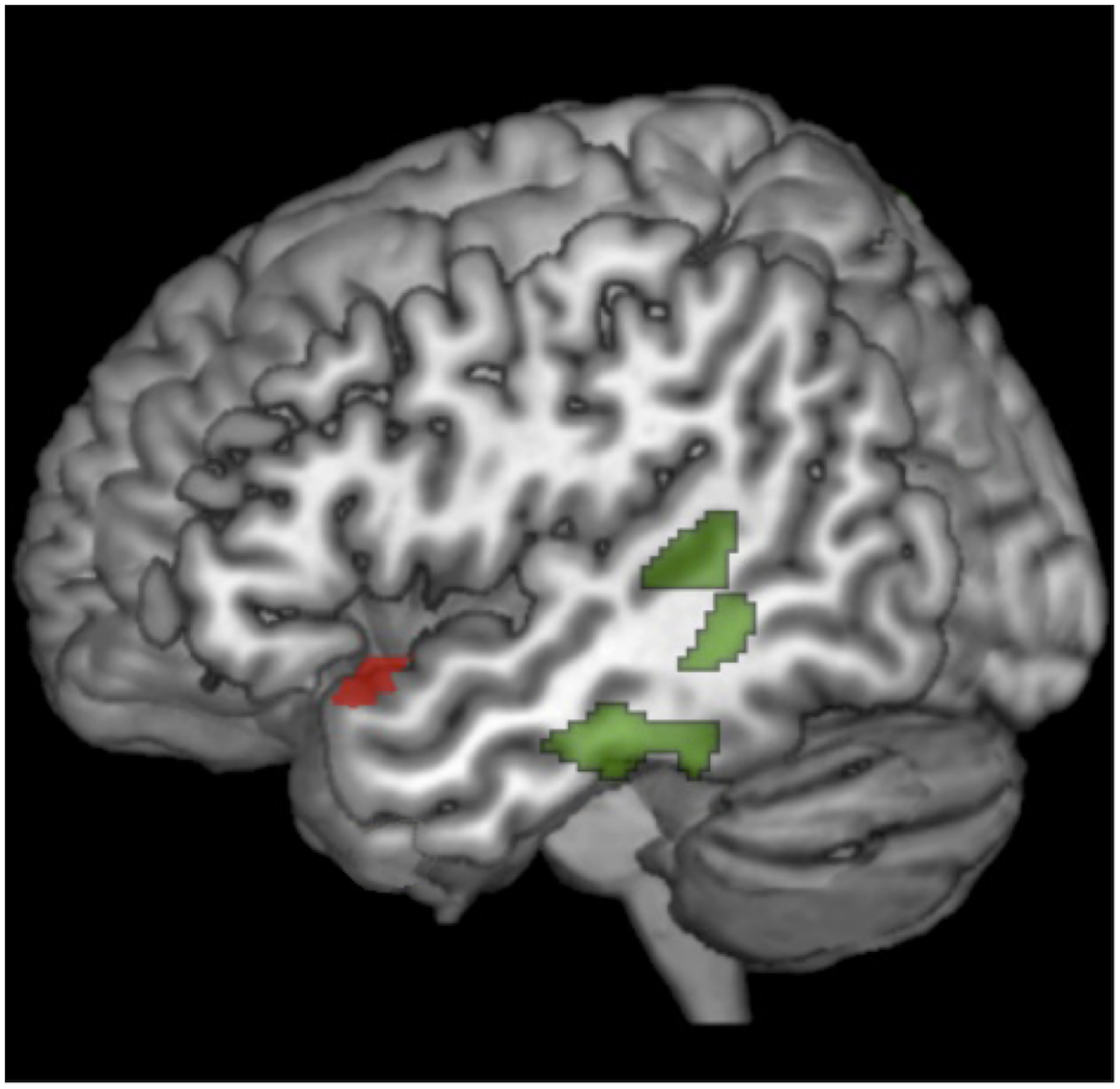
Correlation of gray matter volumes with performance on naming for each PPA variant: lv-PPA (green), sv-PPA (red), nfv-PPA (no regions). Results are superimposed on the 3-dimensional rendering of the Montreal Neurological Institute standard brain.

In the first series of analyses, we explored cortical correlations of naming scores for each PPA subgroup to identify areas impacting the naming process in the different PPA variants and to disentangle the core regions of the lexical/semantic word finding network. In sv-PPA, picture-naming scores positively correlated with superior portions of left superior temporal pole. In lv-PPA naming performance positively correlated with the left posterior superior/middle temporal cortex and the inferior temporal cortex. Smaller clusters were found in the inferior frontal and the lingual gyri (p < 0.001, uncorrected). No correlation with naming scores was found for nfv-PPA. Furthermore, no correlation was found for scores of ‘single-word-comprehension’ in these segregated analyses. The results are summarized in Table 4 and illustrated in Fig 2.

**Table 4:** Correlation between gray matter regions and performance with picture naming and single-word comprehension.

Cortical region (Brodmann area)	Coordinates (x, y, z)	T value	Z score	*Cluster size*
***Picture Naming***	//////////////////////	/////////	////////	/////////
**Sv-PPA**	//////////////////////	/////////	////////	*/////////*
Left superior temporal pole (38)	-44, 21, -12	14.51	4.50	*149*
**Lv-PPA**	//////////////////////	/////////	////////	
Left superior/middle posterior temporal gyrus (42, 21)	-50, -43, -6. -52, -37, 18	6.90. 5.67	3.68. 3.37	*112*. *99*
Left inferior temporal gyrus (20)	-46, -24, -24	11.18	4.41	*171*
Left inferior frontal gyrus (45)	-34, 32, 16	6.43	3.57	*108*
Left lingual (18)	-15, -81, -2	11.18	4.41	*112*
**All PPA**	//////////////////////	/////////	////////	*/////////*
Left superior-middle temporal gyrus (38, 21)	-34, 8, -28. -42, 15, -17	7.38. 7.32	5.33. 5.25	*6271*
Left inferior temporal/fusiform gyrus (20)	-40, -28, -20	7.19	5.25	*300*
***Single-word comprehension***	//////////////////////	/////////	////////	*/////////*
**All PPA**	//////////////////////	/////////	////////	*/////////*
Left superior/middle temporal gyrus (21)	-56, 2, -17	3.76	3.21	*1057*
Left posterior inferior temporal/fusiform gyrus (37)	-38, -33, -14	4.12	3.57	*360*
***Naming/comprehension overlap (21*, *22)*. *Anatomical Boundaries*:**	*Anterior*: *-54*, *16*, *-18*. *Posterior*: *-54*, *-6*, *-18 Superior*: *-53*, *7*, *-10 Inferior*: *-51*, *7*, *24*	*///////*	*///////*	*///////*

**Fig 3:**
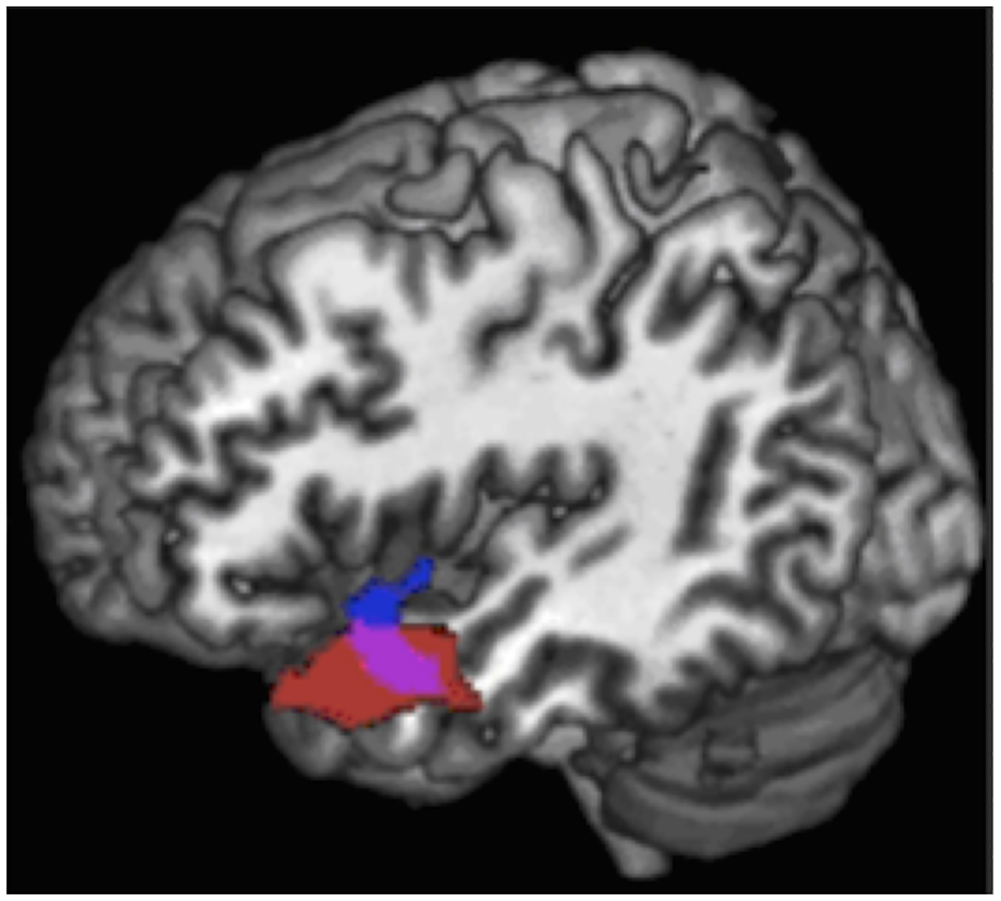
Correlation of gray matter volumes with performance on naming (red) and single-word comprehension (blue) for the whole PPA group. The overlap region representing the lexical-semantic hub is shown in pink. Results are superimposed on the 3-dimensional rendering of the Montreal Neurological Institute standard brain.

In the second series of correlation analyses, we combined the three PPA subgroups with the aim of identifying a distinct region impacting naming in all PPA variants and corresponding to a semantic-to-lexical integration hub. Correlation analyses showed that picture naming scores were correlated with gray matter volume in the left superior-middle temporal gyrus (p < 0.05, FWE). Smaller regions of correlation were also found in the left posterior-ventral part of inferior temporal cortex (p < 0.05, FWE). We then performed a correlation analysis with ‘single-word comprehension’ to identify a common region impacting lexical-to-semantic integration, predicting overlap with the latter correlation result. This analysis showed that scores of single-word comprehension correlated with the left superior/middle temporal gyrus (p < 0.001, uncorrected). Smaller regions of correlation were also found in left inferior temporal gyrus (p < 0.001, uncorrected). Using *mricron* software [31], we compared the correlation regions linked to ‘picture naming’ and ‘single-word comprehension’ and found a unique area of substantial overlap situated in the left superior/middle mid-temporal cortex (MNI boundary coordinates: anterior -54, 16, -18; posterior -54, -6, -18; superior -53, 7, -10; inferior -51, 7, 24; Brodmann areas 38 and 21). The results are summarized in Table 4 and illustrated in Fig 3.

## Discussion

We explored the lexical/semantic network of naming in PPA to individualize the cortical regions impacting the naming process in PPA variants, to disentangle the core regions of word finding operations, and to identify a potential hub linking semantic to lexical information. Our findings show that distinct anterior and posterior temporal cortices impact naming in sv-PPA and lv-PPA, respectively, suggesting anatomically dissociated substrates for semantic and lexical processing during word finding. In contrast, naming impairments in nfv-PPA were rare and not linked to any specific cortical correlate. Subsequent analyses combining the three variants unveiled the existence of a third key area, central to word finding in PPA, which might correspond to a bidirectional ‘hub’ linking semantic and lexical word information located in the middle and superior mid-temporal cortex.

More specifically, our behavioral results showed that aphasia severity was similar in the three PPA variants but that naming scores were distinct, with lowest performance in sv-PPA followed by lv-PPA and nfv-PPA. The correlation analyses indicated that naming difficulties in sv-PPA were related to superior regions of the left temporal pole whereas, in lv-PPA, they were linked to three contiguous regions including the left posterior superior, posterior middle and middle inferior temporal cortex. VBM analyses of the anatomical patterns differentiating PPA variants demonstrated that the identified anterior and posterior cortices for naming corresponded to regions specifically damaged in sv-PPA and lv-PPA respectively, confirming the anatomo-functional separation of word finding operations. However, our results also highlight that critical zones for naming involve zones beyond the most atrophied regions such as the middle inferior temporal cortex in the lv-PPA variant. They also specify the precise naming correlates within the most atrophied regions thus providing information about the exact locus primarily involved in naming capacities in the lv-PPA and the sv-PPA variants.

As discussed below posterior temporal regions might play a causative role in generating lexical information whereas the integrity of anterior temporal regions would be necessary for the processing of semantic information. Subsequent analyses combining the three PPA variants showed that naming correlated with an anatomically distinct area in middle/superior mid-temporal cortices which plays an additional core role for word finding. A second analysis with single-word comprehension revealed a similar left temporal region with considerable anatomical overlap, suggesting its role in the bidirectional integration of lexical/semantic information during word production and word comprehension. Anatomical analysis of this integrative and bidirectional ‘overlap zone’ confirmed its central localization in superior/middle mid-temporal cortices. These findings combining the three variants also highlight that central correlates of naming in PPA as such, and the integrative overlap region, are outside of the most atrophied regions but that they critically impact naming capacities and integrative word processing. These temporal-cortical regions are slightly more caudally and rostrally located as compared to the most atrophied regions and naming correlates in sv-PPA and lv-PPA, respectively. Furthermore, it should be noted that, in the entire PPA group, the correlate of single-word comprehension did not completely overlap with the correlate of naming. Functionally, naming accuracy depends on previous access to semantic information of a given word as evidenced in sv-PPA and a computational model by Lambon Ralph et al. [32]. However, our anatomical results indicate that the serially different steps of word processing are implemented by distinct but tightly interrelated neural populations. The entire region (encompassing the blue and red marked areas in Fig 3) is involved in single-word processing, yet with differential contributions. The blue area (Fig 3) appears to be more dedicated to single-word comprehension and semantics while the red area interacts at the stage of lexical access for naming. These differential roles are reflected by slightly distinct correlation areas which share, however, a great overlap zone. This overlap zone appears to integrate the distinct contributions of word processing while binding semantic comprehension and lexical word-production information required for subsequent naming.

### Naming in PPA and brain implementation of word finding

Word finding and naming difficulties have been reported to be the single most common language difficulty in PPA [1]. Our data indicate that this claim must be put into perspective given that naming disorders show a highly unequal distribution across PPA variants. Naming impairment is more important in sv-PPA than in lv-PPA (but affecting in both 100% of the patients), and is rare and slight in nfv-PPA (19% of the patients), which at the group level had normal scores with the naming task. This differential pattern within the PPA spectrum is consistent with previous studies for which a detailed inspection of naming scores reveals a similar inter-variant naming gradient [11, 14, 24]. Although naming scores are not considered to be a good discriminator between PPA variants [24] normal performance with sensitive naming tests for a given patient appears to rule out sv-PPA and lv-PPA, but not nfv-PPA diagnosis. Such normal or only mildly hampered performance in nfv-PPA can be explained by damage to post-lexical stages of word production impacting phoneme concatenation during phonological encoding [33] but leaving unaffected the lexical/semantic core processes. This damage pattern is also consistent with our findings of predominant phonemic paraphasias in nfv-PPA as compared to lv-PPA or sv-PPA.

The cortical regions impacting naming in PPA variants have been investigated only by few studies suggesting a major role for anterior/middle/posterior regions of the lateral temporal cortex. However, these studies intermingled PPA with other neurodegenerative conditions [15, 16], combined PPA variants into a unique PPA group [14] or did not explore the whole range of PPA variants [6]. The question of cortical naming correlates in the three PPA variants therefore remained an open issue. The present study fills this gap specifying that damage to posterior temporal regions modulates naming in lv-PPA whereas damage to superior portions of the temporal pole impacts performance in sv-PPA. These findings are consistent with reported atrophy patterns in lv-PPA and sv-PPA [11] and they reveal, within these atrophic regions, the specific brain areas impeding the naming process in both variants. By contrast, no region was identified for nfv-PPA presumably due to the rare, slight and poorly varying naming deficits in this subgroup. This finding is also related to our performance rating procedure focalising explicitly on the lexical/semantic core of word finding and counting as correct post-lexical errors of phonological encoding when target words were recognizable (e.g., ‘edephant’ or ‘edepant’ for ‘elephant’). Relatively preserved naming might also result from the fact that we specifically assessed nouns but not verb naming which has been shown to be altered in nfv-PPA [34]. It should however be noted that verb naming is a more demanding task which might not only depend on lexical-semantic core functions but crucially involve executive capacities enabling the identification and extraction of the actions performed on the test pictures.

Regarding the linguistic function of the temporal regions evidenced here, the literature on PPA suggests that anterior regions of the temporal cortex are involved in semantic processing whereas the posterior regions might impact lexical processing. This view is consistent with the fact that lv-PPA patients have impaired naming but relatively spared semantic abilities [11, 35] whereas sv-PPA patients have both verbal and nonverbal semantic deficits [36, 37]. However, several authors have proposed that sv-PPA patients also have lexical impairments [38] and that lv-PPA patients also demonstrate semantic deficits [13, 29], making anatomical lexical/semantic distinctions difficult. A second line of evidence for word-finding correlates derives from studies with vascular patients showing that lexical processing involves various mainly left sided cortical regions comprising primarily posterior temporal cortices [39–41]. Such studies are consistent with our naming correlates in lv-PPA but they are blind with respect to the temporal pole given that stroke rarely causes damage to anterior regions of the temporal lobe [9, 10, 39].

The perhaps most important evidence for lexical/semantic substrates derives from functional imaging studies with experimental paradigms specifically tapping either the lexical or semantic level through the use of implicit processing tasks which minimize conscious control processes and inter-level interference. Graves et al. [42] used priming with nonce word stimuli that by definition have no semantic representation but that were progressively lexicalised through repeated stimulus exposure. Functional MRI with healthy adults pronouncing the target stimuli showed specific implication of left superior-posterior temporal cortex, which is concordant with our naming correlates for the lv-PPA group. Likewise, the orthogonal manipulation of lexical frequency and concept familiarity of target words in a picture naming task showed that frequency effects were specifically related to posterior temporal cortices [43]. These results were replicated via a similar paradigm confirming that lexical frequency, but not semantic familiarity, correlated with the MRI signal in superior and inferior posterior cortices [44], encompassing the major regions identified by our data in lv-PPA. Implicit processing tasks were also used to explore correlates for semantic processing including masked semantic priming. Such unconscious priming experiments are a particularly strong test to assess whether automatic semantic processing is genuinely related to anterior temporal regions as initially suggested by studies with sv-PPA [11, 45]. In a functional MRI study with healthy adults Lau et al. [46] reported effects of masked semantic priming related to exactly the same cortical region as evidenced in our sv-PPA group: the superior portion of the temporal pole. These latter finding is consistent with various accounts stipulating that the temporal pole represents a central region for semantic processing [5], and it specifies that only its superior portion is relevant for the funnelling of semantic information into word finding processes.

The anterior/posterior distinction of semantic/lexical processing reflected by these studies and reinforced by our data has received additional support from a functional MRI study with healthy adults assessing semantic matching of famous faces, and comparing trials with proper name retrieval and trials without name retrieval [3]. Lexical retrieval of names was linked to superior-posterior and mid-posterior temporal cortices as in our lv-PPA group whereas semantic processing was related to bilateral temporal poles. Altogether, the combination of previous findings with our data shows that the word finding process involves a neural network comprising two critical core regions within the temporal cortex: the superior temporal pole for the processing of word-related semantic information and posterior temporal cortices for the activation of the lexical word form. This anterior/posterior polarity is highlighted in the present study on a large PPA population which also provides causative evidence that damage to these temporal regions necessarily leads to naming disorders: lexical anomia in lv-PPA and semantically induced anomia in sv-PPA.

### The lexical-semantic hub

Our findings combining the three PPA variants have shown that the anterior/posterior polarity is bridged by an intermediate region in middle and superior mid-temporal cortices. This region seems central to naming in all variants and it presumably implements a key step in the word finding process. Most models of word production assign a key role to a processing step underpinning the linkage of semantic and lexical information, but the terminology designating this intermediate stage and the related representations is confusingly variable, including ‘lexical selection’ and ‘lemma representations’ [2, 17], ‘modality-independent lexical access’ and ‘lexical-semantic features’ [39] or ‘dispositions of convergence zones’ [7, 8]. Given that this intermediate step is distinct from lexical and semantic processing several authors have suggested that the underlying substrate would be situated between posterior lexical and anterior semantic regions of the temporal lobe [39, 44]. Our correlations with the combined PPA group evidenced such an anatomically intermediate and distinct region which specifically involves mid-temporal cortices. This finding is also consistent with results of Damasio et al. [7] showing that picture naming involves category-specific regions (e.g., ‘persons’, ‘tools’, ‘animals’) but that combining all categories yielded activation in a unique circumscribed region posterior to the temporal pole. Similar anatomical results were also obtained by Hurley el al. [47] who showed that the access to precise and detailed lexical semantics, as opposed to general concepts, depends on a cortical region involving primarily the superior temporal gyrus in its middle and anterior portions, thus providing supplementary support for our findings.

In addition to the central localization, an integrative ‘lexical-semantic hub’ is also expected to implement a particular functional property, namely the computational capacity to link lexical and semantic information independently from the processing modality, i.e. word production or perception. Such a bidirectional function, mapping semantic-to-lexical and lexical-to-semantic information, was explicitly claimed by several authors who stipulated the existence of such an intermediate processing level [2, 7, 17]. We checked this bidirectional binding function through comparing anatomical correlates related to naming and to single-word comprehension. The comparison revealed a region of considerable anatomical overlap for both tasks identifying the lexical-semantic hub in superior and middle mid-temporal regions of the left temporal cortex. Our correlation analyses with behavioral data from three tasks involving lexical and semantic processing in both, word production and perception, addressed a directly related issue. They showed a triple correlation between ‘naming’, ‘single-word comprehension’ and ‘category fluency’ indicating a unique functional substrate that presumably corresponds to the anatomical mid-temporal hub.

We finally compared the coordinates of the identified hub region with two influent models of word processing [2, 48]. The first model [2] derived from a meta-analysis on word production claims that the lemma level mediating between semantic and lexical representations is localized in the middle mid-temporal cortex. The coordinates of our lexical-semantic hub overlap this cortical region. Similarly, the anatomical model for single word processing of Hickok and Poeppel [48] claimed that the ‘sound-meaning interface’ linking phonological codes to semantics essentially involves the ‘middle temporal gyrus’ in its posterior and mid-temporal portions. The lexical-semantic hub proposed here is consistent with this claim and further specifies the crucial role for the mid-temporal portion.

In addition to such evidence for a mid-temporal hub one might ask whether its brain correlates correspond to available reports of anatomical regions underpinning naming performance. Most authors have shown that the multiple-step process of naming depends on a large-scale network including temporal, occipital, parietal and frontal regions [6], which impact processes of visual picture identification up to late operations of phonological encoding and word articulation. However, studies focusing on the semantic-lexical core of the word finding process have delineated lateral temporal cortices as the essential contributors. An important number of these studies have reported data reflecting the involvement of superior and middle mid-temporal regions corresponding to the coordinates of the hub identified by our results [7, 38, 49–52].

## Conclusion and Limitations

Our findings indicate that several core regions impact naming in PPA variants: the superior temporal pole in sv-PPA and posterior temporal cortices in lv-PPA, dedicated to semantic and lexical processing aspects, respectively. In addition, a central hub anatomically distinct from the two previous regions and located in the mid-temporal region appears to play an essential role in naming and word processing, in that it enables the integrative and bidirectional binding of semantic and lexical information. These results are important for both anatomical models of language processing and clinical issues in PPA. Firstly, they indicate an anterior-posterior temporal axis for word finding operations and suggest the cortical coordinates of the lexical-semantic integration hub in the mid-regions of this axis. Further studies are however required for exploring the existence of white matter connections between these temporal core regions and investigating their functional cross-talk. Although several authors have shown the role of long-distance connections in PPA language dysfunction [53–57] anatomo-functional studies on local connectivity within the temporal lobe are lacking. Likewise, further studies are needed to confirm the coordinates of the proposed hub by using linguistic tasks specifically tapping lexical processes, semantic representations and intermediate lemma formation. Secondly, our findings provide information relevant to clinical practice by indicating the underlying mechanisms and the anatomical characterization of anomia in PPA. With the prospect of future therapy, they might be of considerable interest for linguistically-driven anomia rehabilitation and for transcranial stimulation trials focusing on word-related target regions in aphasic patients. Furthermore, identification of atrophy in the lexical-semantic hub region might be of interest for differentiating nfv-PPA from lv-PPA or sv-PPA. In particular, distinguishing cases of lv-PPA and nfv-PPA can be linguistically challenging and MRI findings in single subjects are often difficult to evaluate by eye, especially for lv-PPA and nfv-PPA. The presence of early damage in the lexical-semantic hub in lv-PPA and its absence in nfv-PPA could eventually be a useful MRI biomarker for differential diagnosis. This perspective requires and encourages the development of reliable volumetry/morphometry techniques targeting the superior-middle mid-temporal hub region at the individual level.

The present study has several limitations that encourage the replication of our findings in larger PPA cohorts with additional well-controlled experimental materials. A first limitation is that the stimuli for the naming and the single-word comprehension task were not perfectly matched for frequency, number of letters, conceptual familiarity and visual picture complexity. Although we deliberately used standard tests to provide global and representative markers of patient performance in both domains it would be important to prospectively replicate our results with matched stimuli for both tasks to provide directly comparable conditions for naming and word comprehension. A second limitation might be the inclusion of some left-handed patients (one out of twelve lv-PPA patients and two out of eleven sv-PPA patients), which could have induced induce slight biases with respect to the anatomical lateralization of our correlation data. However, such potential biases appear to be not substantial given a recent study of Miller et al. [58] who have investigated the contribution of left-handedness in PPA. The authors concluded that even if this condition is relatively frequent the right-handed and left-handed cohorts were homogeneous on imaging, cognitive profiles, and structural analysis of brain symmetry. It should also be noted that the cortical thickness comparisons between our three PPA groups showed that lv-PPA and sv-PPA (containing a very weak proportion of left-handers) demonstrated the ‘classical’ strongly left-sided atrophy pattern. Third, the right-predominant pattern of atrophy in our nfv-PPA group could be misleading although several studies have shown right frontal atrophy in nfv-PPA [59, 60]. An explication of this right-lateralization is linked to the fact we analyzed atrophy patterns by direct comparisons between the three PPA groups with the aim to evidence group-specific atrophy patterns. Overlap of atrophy patterns in these groups (e.g., between nfv-PPA and sv-PPA/lv-PPA), with possibly extensive atrophy patterns in lv-PPA and sv-PPA, implies that left frontal regions become less represented in nfv-PPA. For the same reason, the direct comparisons have disclosed areas in the right hemisphere, which are usually less evident. However, a limitation of the present study is that we did not compare each PPA group with healthy controls which might have revealed the ‘classical’ more extensive left frontal atrophy in nfv-PPA. Finally, another potential limitation requiring replication of our results is that we included two lv-PPA patients with slight single-word comprehension difficulties, which is not at odds with a lv-PPA diagnosis [29, 30], but which might have favored an slight over-representation of semantic dysfunction in our entire PPA group. As a consequence our results might have slightly overestimated the size of the word-comprehension correlate and the area of the ‘lexical-semantic hub’. We however are confident that we provided a valuable delineation of the hub region which opens new perspectives for replicating and extending our results with large, homogenous cohorts of aphasia patients.

## Supporting Information

S1 Dataset Raw demographic, cognitive and language data of the thirty PPA patients. (PDF)
