# Supplementary material for: The Brain Network of Naming: A Lesson from Primary Progressive Aphasia
Source: PLoS One. 2016 Feb 22;11(2):e0148707. doi: 10.1371/journal.pone.0148707 (PMC4764674; doi:10.1371/journal.pone.0148707)
Supplement: S1 Dataset — (PDF) [file pone.0148707.s001.pdf]

subject	PPA variant	Man/Woman	age	years education	handedness	symptom duration
subj1	nfv-PPA	W	71	6	R	4
subj2	nfv-PPA	W	64	6	R	4
subj3	nfv-PPA	W	80	12	R	3
subj4	nfv-PPA	M	65	8	R	1
subj5	nfv-PPA	M	75	17	R	2
subj6	nfv-PPA	W	66	12	R	5
subj7	nfv-PPA	W	71	8	R	3
subj8	lv-PPA	W	80	17	R	3
subj9	lv-PPA	M	76	17	R	3
subj10	lv-PPA	W	81	5	R	3
subj11	lv-PPA	W	77	8	R	3
subj12	lv-PPA	W	70	8	R	2
subj13	lv-PPA	M	65	8	R	3
subj14	lv-PPA	M	59	12	L	3
subj15	lv-PPA	M	60	9	R	3
subj16	lv-PPA	M	72	9	R	3
subj17	lv-PPA	M	64	11	R	4
subj18	lv-PPA	M	58	8	R	4
subj19	lv-PPA	M	65	11	R	4
subj20	sv-PPA	M	64	8	L	2
subj21	sv-PPA	W	54	10	L	4
subj22	sv-PPA	M	52	19	R	3
subj23	sv-PPA	M	66	10	R	3
subj24	sv-PPA	M	67	9	R	3
subj25	sv-PPA	W	62	14	R	2
subj26	sv-PPA	W	66	9	R	2
subj27	sv-PPA	M	57	10	R	7
subj28	sv-PPA	M	76	17	R	2
subj29	sv-PPA	W	62	8	R	5
subj30	sv-PPA	W	63	7	R	4

DO80	DO 80 Non responses	DO 80 Paraph phonem	DO 80 Paraph sem	phonetic distorsions
65	4	8	0	12
75	0	4	0	8
77	1	5	1	11
77	0	4	0	14
78	1	2	1	6
79	0	0	0	5
80	0	2	0	9
32	6	0	0	0
43	33	0	4	0
52	20	0	5	0
53	20	3	4	0
53	21	0	5	0
58	13	1	7	0
70	6	0	4	0
71	0	3	6	0
73	1	2	5	0
75	1	0	4	0
75	4	0	1	0
75	2	1	2	0
6	59	0	15	0
12	49	1	13	0
24	5	2	11	0
25	34	0	21	0
34	23	0	23	0
38	20	0	22	0
43	4	2	26	0
53	9	0	18	0
56	12	0	12	0
61	11	1	7	0
67	10	0	3	0

single-word compreh	fluency 'fruits'	fluency 'P'	sentence repetition	syntax Leyton	MMSE
71	18	2	7	2	27
71	10	13	6	3	23
72	17	24	13	2	29
71	9	1	8	2	25
71	14	10	6	3	25
70	22	11	4	2	27
73	19	14	4	3	27
53	5	2	11	0	7
69,5	4	9	12	0	12
70	6	8	9	0	24
70	10	5	4	0	21
71	2	5	7	0	17
69	10	8	4	0	19
72	10	14	7	0	21
56,0	5	6	6	0	18
71	11	2	9	0	22
68	11	13	8	0	21
72	11	6	15	1	20
72	14	16	11	0	26
43,5	2	7	12	0	23
35,5	8	13	12	0	22
54,5	5	5	14	0	24
59	4	6	13	0	25
55,5	4	5	15	0	26
62	11	18	16	0	29
51	10	5	15	0	24
69	7	15	15	0	28
67	6	19	13	0	25
66	14	10	14	0	22
68,5	11	9	5	0	23

FAB	aphasia severity (BDAE)
13	2
10	4
18	4
12	3
12	2
15	4
15	3
8	2
7	3
16	3
11	3
10	4
5	3
11	4
12	3
10	3
9	3
13	5
12	4
9	3
12	3
10	4
14	4
12	5
16	3
10	5
17	5
17	3
12	4
13	3
